# Brian 2 - the second coming: spiking neural network simulation in Python with code generation

**DOI:** 10.1186/1471-2202-14-S1-P38

**Published:** 2013-07-08

**Authors:** Marcel Stimberg, Dan FM Goodman, Victor Benichoux, Romain Brette

**Affiliations:** 1 Institut d'Études Cognitives, École Normale Supérieure, Paris, 75005, France; 2 Laboratoire de Psychologie de la Perception, CNRS and Université Paris Descartes, Paris, 75006, France; 3 Department of Otology and Laryngology, Harvard Medical School, Boston, Massachusetts, 02114, USA; 4 Eaton-Peabody Laboratories, Massachusetts Eye and Ear Infirmary, Boston, Massachusetts, 02114, USA

## 

Brian 2 is a fundamental rewrite of the Brian [1,2] simulator for spiking neural networks. Brian is written in the Python programming language and focuses on simplicity and extensibility: neuronal models can be described using mathematical formulae (differential equations) and with the use of physical units. Depending on the model equations, several integration methods are available, ranging from exact integration for linear differential equations to numerical integration for arbitrarily complex equations. The same formalism can also be used to specify synaptic models, allowing the user to easily define complex synapse models.

Brian 2 keeps most of the syntax and functionality consistent with previous versions of Brian, but achieves more consistency and modularity as well as adding new features such as a simpler and more general new formulation of refractoriness. A consistent interface centered around human-readable descriptions using mathematical notation allows the specification of neuronal models (including complex reset, threshold and refractory conditions), synaptic models (including complex plasticity rules) and synaptic connections. Every aspect of Brian 2 has been designed with extensibility and adaptability in mind, which, for example, makes it straightforward to implement new numerical integration methods.

Even though Brian 2 benefits from the ease of use and the flexibility of the Python programming language, its performance is not limited by the speed of Python: At the core of the simulation machinery Brian 2 makes use of fully automated runtime code generation [3], allowing the same model to be run in the Python interpreter, in compiled C++ code or on a GPU using CUDA libraries[4]. The code generation system is designed to be extensible to new target languages and its output can also be used on its own: for situations where high performance is necessary and/or where a Python interpreter is not available (for example for robotics applications), Brian 2 offers tools to assist in assembling the generated code into a stand-alone version that runs independently of Brian or a Python interpreter.

To ensure the correctness and maintainability of the software, Brian 2 includes an extensive, full coverage test suite. Debugging of simulation scripts is supported by a configurable logging system, allowing simple monitoring of the internal details of the simulation process.

Brian is made available under a free software license and all development takes place in public code repositories [5].
